# Low resting heart rate, sensation seeking and the course of antisocial behaviour across adolescence and young adulthood

**DOI:** 10.1017/S0033291717003683

**Published:** 2018-01-09

**Authors:** Gemma Hammerton, Jon Heron, Liam Mahedy, Barbara Maughan, Matthew Hickman, Joseph Murray

**Affiliations:** 1 Population Health Sciences, University of Bristol, Bristol, UK; 2 MRC Social, Developmental and Genetic Psychiatry Centre, Institute of Psychiatry, Psychology & Neuroscience, King's College London, London, UK; 3 Postgraduate Program in Epidemiology, Universidade Federal de Pelotas, Pelotas, Brazil

**Keywords:** ALSPAC, antisocial behaviour, heart rate, mediation, sensation seeking

## Abstract

**Background:**

Low resting heart rate (RHR) is a consistent biological correlate of antisocial behaviour (ASB), however potential mechanisms have been largely unexplored. We hypothesise that lower RHR will be associated with higher ASB levels in mid-adolescence and persistence into adulthood, and that these associations will be explained, in part, by sensation seeking and callous-unemotional traits.

**Methods:**

ASB was assessed repeatedly with young people from ages 15 to 21 years in a population-based birth cohort (ALSPAC). A longitudinal trajectory was derived and showed ASB decreasing across adolescence before stabilising in early adulthood. RHR was recorded at age 12 years, and mediators were assessed at age 14 years.

**Results:**

After adjusting for socio-demographic confounders, there was evidence for a total effect of RHR on ASB levels in mid-adolescence [b(95% CI) = −0.08 (−0.14 to −0.02)], reflecting 0.08 more types of antisocial activity in the last year per 10 fewer heart beats per minute. This effect was almost entirely explained through sensation seeking [b(95% CI) = −0.06 (−0.08 to −0.04)]. After additionally adjusting for child and parent-related confounders, all effects weakened; however, there was still evidence of an indirect effect of RHR, via sensation seeking, on ASB levels in mid-adolescence [b(95% CI) = −0.01 (−0.03 to −0.003)]. There was no evidence for a total effect of RHR on ASB levels in early adulthood, and weak evidence of an indirect effect, via sensation seeking [b(95% CI) = −0.01 (−0.01 to −0.00)].

**Conclusions:**

Lower RHR in childhood was associated with higher ASB levels in mid-adolescence, indirectly via sensation seeking.

## Introduction

The age-crime curve consistently shows that antisocial behaviour (ASB) peaks in mid-adolescence and then declines throughout late adolescence and early adulthood (Moffitt, 1993; Farrington, 1995). However, there is evidence for individual differences in the course of ASB across this time period (Moffitt, 1993), and identifying factors associated with desistance is important to guide post-onset interventions (Kazemian, 2007). Low resting heart rate (RHR) is one of the most consistent biological correlates of ASB, with meta-analyses highlighting that the relationship is present in both childhood and adolescence (Ortiz & Raine, 2004; Portnoy & Farrington, 2015). Increasing evidence is also emerging from longitudinal studies suggesting that lower RHR is associated with crime levels in adulthood, after adjusting for a wide range of physical, socioeconomic and psychiatric confounders (Jennings et al. 2013; Latvala et al. 2015; Murray et al. 2016). However, it is currently unknown whether heart rate impacts on the course of ASB across adolescence and early adulthood, with the few studies that have examined the relationship between RHR and within-individual changes in ASB showing inconsistent results (Moffitt & Caspi, 2001; Loeber et al. 2007; Baker et al. 2009). For example, in one community sample of males, lower RHR in childhood differentiated those with persistent ASB from those with ASB limited to adolescence (Moffitt & Caspi, 2001); whereas, in another male community sample, lower RHR in adolescence did not predict ASB persistence (Loeber et al. 2007). Additionally, a more recent longitudinal twin sample showed no association between lower RHR at age 9 years and change in ASB between ages 9 and 14 years (Baker et al. 2009). Therefore, more research is needed to examine the biological underpinnings of desistance in order to identify physiological factors that could be used to screen for those at risk for persistent ASB or to guide post-onset interventions (Kazemian, 2007; Loeber et al. 2015).

A number of theoretical explanations have been proposed to explain the association between low RHR and ASB (Ortiz & Raine, 2004; Portnoy & Farrington, 2015), of which sensation seeking theory has received the most support. This theory suggests that low RHR is a marker of low autonomic arousal, and therefore may be an unpleasant physiological state which leads those with a low heart rate to seek out stimulating activities, including ASB, in order to increase their autonomic activity to more optimal levels (Raine, 2002). Two studies have shown that sensation seeking mediates the association between lower heart rate and ASB (Sijtsema et al. 2010; Portnoy et al. 2014). Using a Dutch general population sample, Sijtsema et al. (2010) found that sensation seeking mediated the association between lower RHR at age 11 years and rule-breaking at age 16 years in boys, but not girls. More recently, in a cross-sectional sample of boys aged 16 years, it was found that sensation seeking mediated the association between lower heart rate and both aggression and nonviolent delinquency (Portnoy et al. 2014). These studies were among the first to provide evidence for the importance of sensation seeking as a mechanism, however, there are a number of shortcomings to consider. These include the use of a cross-sectional design (Portnoy et al. 2014), which can result in biased estimates for longitudinal parameters in mediation analyses (Maxwell & Cole, 2007), and the lack of adjustment for important physical and psychiatric confounders (such as body mass index, fitness, alcohol consumption, and smoking) that may be associated with both cardiovascular functioning and ASB (Sijtsema et al. 2010).

Additionally, more recent research suggests that the association between lower RHR and ASB may also be explained through a broader range of traits associated with under-arousal including callous-unemotional traits and psychopathy (DeLisi, 2009; Portnoy & Farrington, 2015; Kavish et al. 2017). For example, a previous cross-sectional study found an association between RHR and callous-unemotional traits in male adolescents (Kavish et al. 2017), and a recent meta-analysis reported evidence of an association between RHR and psychopathy (Portnoy & Farrington, 2015). In turn, psychopathic traits can accentuate ASB through adolescence and early adulthood (DeLisi, 2009). Finally, no studies that we are aware of have examined potential mediators of the association between lower heart rate and the course of ASB across adolescence and early adulthood.

The present investigation uses a large, prospective population cohort to examine the association between lower RHR in childhood and the course of ASB from adolescence into adulthood, and to investigate whether any association found is mediated by sensation seeking or callous-unemotional traits. The primary hypothesis is that lower RHR will be associated with higher levels of ASB in mid-adolescence, and that this association will be explained, in part, by both sensation seeking and callous-unemotional traits. The secondary hypothesis is that lower RHR will also be associated with slower decline and higher levels of ASB in adulthood.

## Method

### Sample

Data were utilised from a large UK birth cohort; the ‘Avon Longitudinal Study of Parents and Children’ (ALSPAC) which was set up to examine genetic and environmental determinants of health and development (Boyd et al. 2013). The ‘core’ enrolled sample consisted of 14 541 pregnant women resident in the former county of Avon, UK, who had an expected date of delivery between 1 April 1991 and 31 December 1992. Of the 13 988 offspring alive at 1 year, a small number of participants withdrew consent (*n* = 24). The sample was also restricted to singletons or first-born twins, leaving a starting sample of 13 775. Parents and children have been followed up regularly since recruitment via questionnaire and clinic assessments. Further details on the sample characteristics and methodology have been described previously (Boyd et al. 2013; Fraser et al. 2013) and detailed information about ALSPAC can be found on the study website (http://www.bristol.ac.uk/alspac). For information on all available ALSPAC data see the fully searchable data dictionary (http://www.bris.ac.uk/alspac/researchers/data-access/data-dictionary). Ethical approval for the study was obtained from the ALSPAC Ethics and Law Committee and the Local Research Ethics Committees.

### Measures

Data were collected both during focus clinics and via questionnaires that were either returned by post or completed online. A timeline for data collection is shown in online Supplementary Fig. S1.

#### Resting heart rate

During the focus clinic at approximately age 12 years (mean = 11 years 9 months, s.d. = 3 months) blood pressure and pulse rate (beats per minute; bpm) were recorded as part of a 20-minute session using a Dinamap 9301 Vital Signs Monitor (Morton Medical, London, UK) on the right arm while the respondent was seated. Before pulse rate was recorded, the child was made to sit comfortably in a chair and was given a simple explanation of what would happen in the session. The child was encouraged to sit quietly while the measurement was taken and the parent was asked about any medications the child was currently on and when they had last been taken. To minimise random measurement error, a second reading was taken immediately afterwards and a mean was calculated.

#### Antisocial behaviour

A self-report battery of questions asking about antisocial acts committed in the past year was completed by the young person at four time points between ages 15 and 21 years (Smith & McVie, 2003). At age ~15 years (mean = 15 years 6 months, s.d. = 4) and ~18 years (mean = 17 years 10 months, s.d. = 5) data were collected during a computer-based session at a focus clinic and at ages ~19 years (mean = 18 years 8 months, s.d. = 6) and ~21 years (mean = 20 years 11 months, s.d. = 6) data were collected via online or postal questionnaire. Eight ASB items were consistent across all time points (stole from shops, broke into a vehicle or building, stole from a person, damaged property, assault, carried a weapon, rowdy in a public place, hurt animals). All items were then combined to create a sum score representing a total number of types of crime committed in last year at each time point (range: 0–8). External validity for this self-report questionnaire has been examined previously in adolescents using cross-checks with agency records and teachers’ questionnaires (Smith et al. 2001).

#### Potential mechanisms

##### Sensation seeking

Sensation seeking was assessed at approximately age 14 years (mean = 13 years 10 months) during a focus clinic with the young person. Nine items from the intensity seeking subscale of Arnett's Inventory of Sensation Seeking (AISS; Arnett, 1994) were used (the item: ‘in general I work better under pressure’ was not included because it was not thought to be age-appropriate). Previous studies have shown this scale to have good internal reliability, criterion-related validity and construct validity in relation to a wide range of reckless behaviours in adolescence (Arnett, 1994, 1996). During a computer-based session, young people were asked to report whether each item was: ‘very like me’, ‘quite like me’, ‘not much like me’ or ‘not at all like me’. Confirmatory factor analysis (CFA) was used to estimate a unidimensional latent variable for sensation seeking. The factor loading for the first item was fixed to unity to set the scale of the latent variable to be equivalent to the item scale. Sensation seeking items and factor loadings are listed in online Supplementary Table S1a. Higher scores on the latent variable represent increased sensation seeking. The scale showed good internal consistency [ordinal omega (*ω*) = 0.74].

##### Callous-unemotional traits

Callous-unemotional traits were assessed at approximately child age 14 years (mean = 13 years 10 months) using a mother-report questionnaire measure (Moran et al. 2008). Six items were chosen on the basis of factor analyses of scales measuring callous-unemotional traits (Frick et al. 2000), and mothers were asked to report the frequency of each item for their study child with response options: ‘not at all’, ‘rarely’, ’sometimes’, ‘often’, ‘always’. CFA was used to estimate a unidimensional latent variable for callous-unemotional traits. The factor loading for the first item was fixed to unity to set the scale of the latent variable to be equivalent to the item scale. Callous-unemotional items and factor loadings are listed in online Supplementary Table S1b. Higher scores on the latent variable represent increased callous-unemotional traits. The scale showed excellent internal consistency [ordinal omega (*ω*) = 0.83].

##### Potential confounders

In mediation analyses, four assumptions are made with respect to confounding. These include no unmeasured confounders for any of the paths and no measured or unmeasured confounder for the association between mediator and outcome which lies on the causal pathway from the exposure. In the current analyses, the same set of factors were assumed to confound all paths, and these were all assessed, or reported to occur either before or simultaneously to the assessment of the exposure.

Maternal questionnaires completed during pregnancy were used to assess housing tenure (owned/mortgaged; privately rented; subsidised housing rented from council/ housing association), maternal level of education (no high school qualifications; high school only; beyond high school), crowding (up to one person per room in house; more than one person per room), and ethnicity (white; non-white).

Child age (in months), body mass index (BMI), diastolic blood pressure, and any medication use were recorded during the focus clinic at age 12 years. Frequency of vigorous activity was assessed twice with the mother when the child was approximately age 11 years using the question: ‘in the past month, what was the average number of times that your son/daughter participated in vigorous physical activity (such as running, dance, gymnastics, netball, swimming, or aerobics)? Response options ranged from ‘none’ to ‘daily’ and a mean of both time points was taken. During the focus clinic at approximately age 13 years, young people reported the age at which they had their first whole drink of alcohol and the age at which they first smoked a cigarette. Two binary variables were created to represent drinking alcohol or smoking a cigarette before age 11 years. These factors were considered to be important confounds of the associations tested, given that they may influence cardiovascular functioning, sensation seeking, callous-unemotional traits and risk for ASB.

Finally, parental crime and problematic alcohol use were measured on eight occasions from the child's birth to 11 years with questionnaires sent to mothers and their partners asking whether either had occurred since the last assessment. Any positive endorsement from either parent of being in trouble with the law/convicted was coded as positive for parental crime as has been done previously (Murray et al. 2015). Similarly, any report of alcoholism/alcohol problems across the same time period was coded as positive for parental problematic alcohol use. Again, these factors were considered to be confounders, given that they may influence the child's cardiovascular functioning, sensation seeking, callous-unemotional traits and risk for ASB.

### Statistical analyses

#### Latent growth curve for ASB

The longitudinal trajectory for ASB was derived using an exponential decay model in the structural equation modelling (SEM) framework. The traditional exponential decay model was reparametrised to examine three growth factors which were thought to be of interest when examining ASB desistance: the intercept, asymptote, and half-life. The intercept (when fixed at baseline) is the average predicted starting point or initial level of ASB, and the asymptote is a line that the curve approaches as it heads towards infinity, or the average predicted the final level of a person's ASB. The half-life, measured in years, is the time by which 50% of a person's total change in ASB has been observed. Hence, these three parameters characterise an adolescent's starting level of ASB, the rate at which ASB declines, and the expected final level of ASB in adulthood.

An additional parameter was included in the latent growth curve model to allow the trajectory function to absorb artefactual differences between the clinic and questionnaire data collection which may be caused by respondents tending to more readily report antisocial acts in the privacy of a questionnaire assessment completed at home. Subsequently, fit for the ASB trajectory was evaluated by examining residuals for the mean and covariance structure. Further information on the exponential decay model is given in Supplement 1, and has been provided previously (Hammerton et al. 2017).

#### Addressing age variability

To address variability in the age at which respondents completed assessment waves, respondents at each wave were split evenly into ‘younger’ and ‘older’ age groups and these groups were treated as two separate time points in the trajectory analyses. Therefore a total of eight-time points of data were analysed, however, each respondent only contributed a maximum of four pieces of information – akin to an accelerated design (Duncan et al. 1996). The mean ages at each time point were: 15 years 4 months, 15 years 8 months, 17 years 6 months, 18 years 1 month, 18 years 4 months, 19 years 1 month, 20 years 6 months and 21 years 4 months.

#### Estimating the indirect effect of heart rate on ASB via sensation seeking and callous-unemotional traits

There was no evidence for a departure from a linear relationship for RHR and ASB, therefore RHR was treated as continuous in all analyses, and coefficients represent the effect of a change of 10 pulse beats per minute (approximately equivalent to a change of one standard deviation (s.d.) in RHR). Estimates are presented as unstandardised coefficients; therefore, a coefficient of −0.5 for the association between RHR and the ASB intercept would indicate that a 10 bpm decrease in RHR is associated with an increase of 0.5 types of antisocial activities the respondent was involved in, within the last year at age 15 years. A mediation model was run to assess the effect of RHR on ASB growth factors (intercept, half-life and asymptote) via sensation seeking and callous-unemotional traits. The initial model estimated the direct and indirect effects of RHR on ASB whilst allowing residual covariances between ASB growth factors. 95% confidence intervals (CIs) for indirect effects were calculated using the Monte Carlo Method for Assessing Mediation (MCMAM; MacKinnon et al. 2004). Using the parameter estimates for the association between exposure and mediator (path a), and mediator and outcome (path b) and their associated asymptotic (co)variances, a distribution of 20 000 *ab* values was simulated using R v. 3.3.2 (R Core Team, 2015) and a 95% CI around the indirect was calculated (Preacher & Selig, 2012).

All analyses adjusted for socio-demographic confounders (housing tenure, maternal level of education, household crowding index, and ethnicity) to address both potential confounding and selection bias. Potential child-related (sex, age, BMI, blood pressure, medication use, the frequency of vigorous activity, alcohol and cigarette use) and parent-related confounders (crime and alcohol problems) were also taken into account in later analyses. Confounders and RHR were all centred prior to analyses to ensure that growth factors that enter the model non-linearly (i.e. the half-life) maintain a mean of zero (Grimm et al. 2011; Grimm et al. 2013).

#### Secondary analyses

**Fig. 1.**
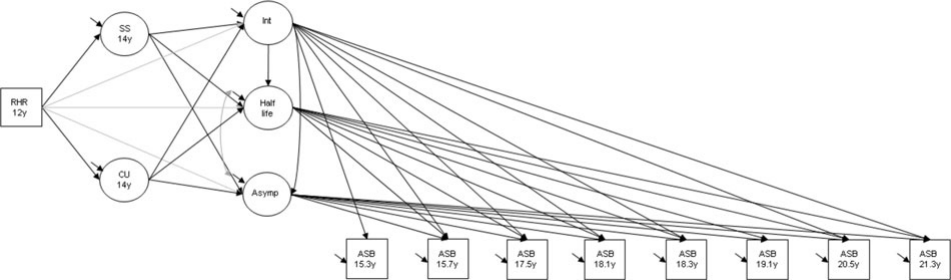
Exponential decay model for ASB showing associations between RHR, sensation seeking, callous-unemotional traits and ASB growth factors; confounders not shown in the diagram; RHR, resting heart rate; SS, sensation seeking; CU, callous-unemotional traits: ASB, antisocial behaviour; Int, intercept; Asymp, asymptote; circles represent latent variables and squares represent observed variables.

In secondary analyses, the model was re-parameterised to examine the direct and indirect paths whilst taking account of the impact of the ASB intercept on the half-life and asymptote. This felt necessary given that the relationship between an exposure (i.e. heart rate) and the rate of decrease in ASB is likely to be dependent on a person's initial ASB level. Additionally, any long-term effects of RHR on ASB levels in early adulthood, are likely to be explained, in part, by the impact of RHR on ASB levels in mid-adolescence. The final model is shown in Fig. 1.

All models were analysed in M*plus* v7.4 (Muthén & Muthén, 2016) using maximum likelihood with robust standard errors (MLR).

### Missing data

Missing data were handled using full information maximum likelihood (FIML) estimation (Enders, 2010). FIML makes the assumption that data are missing-at-random (i.e. given the observed data included in the model, the missingness mechanism does not depend on the unobserved data). This assumption was made more plausible by the inclusion of a number of auxiliary variables, related to missing data. Young people that had complete data for ASB from at least one of four assessments were included in the trajectory analyses (*N* = 6814). The inclusion of confounding factors resulted in a sample size of *N* = 4046 (1837 males and 2209 females). Online Supplementary Fig. S2 shows a flow chart of retention in ALSPAC. Sensitivity analyses were performed using inverse probability weighting (IPW; Seaman & White, 2013). Further information on the IPW analyses is given in online Supplement 2.

## Results

### Descriptive statistics

Descriptive statistics for RHR, ASB, and potential confounders are shown in online Supplementary Table S2, for males and females. Mean RHR at age 12 years was 76 bpm (s.d. = 10.93; 95% reference range = 60–95 bpm). Mean RHR in this sample was slightly lower than population norms for this age group (Fleming et al. 2011; Ostchega et al. 2011).

### Latent growth curve for ASB

**Fig. 2.**
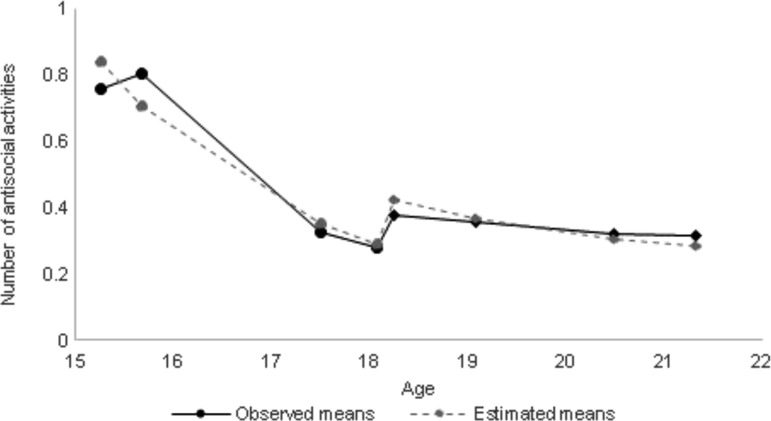
Observed and estimated means for the frequency of ASB: circles represent clinic assessments and diamonds represent questionnaire assessments: *N* = 4046.

Estimated and observed means for the ASB trajectory are shown in Fig. 2 with means, variances, and correlations between growth factors given in online Supplementary Table S3.

The ASB trajectory started at an average of 1.0 (standard error (s.e.) = 0.03) reported antisocial activity per year at age 15 years 4 months and fell to an average of 0.3 (s.e. = 0.03) antisocial activities per year. The mean half-life for ASB was 1 year 6 months (s.e. = 0.17) indicating that, on average, young people reach a halfway point between their initial and final level of ASB at age 16 years 10 months. There was a negative correlation between the intercept and half-life for ASB, indicating that those that had higher initial levels of ASB, approached their final level of ASB more quickly [*r*(s.e.) = −0.59 (0.15), *p* < 0.001]. Additionally, those that had higher initial levels of ASB, had higher final levels (correlation between intercept and asymptote: 0.80 (0.29); *p* = 0.01).

### Estimating the total, direct and indirect effects of RHR on ASB via sensation seeking and callous-unemotional traits

**Fig. 3.**
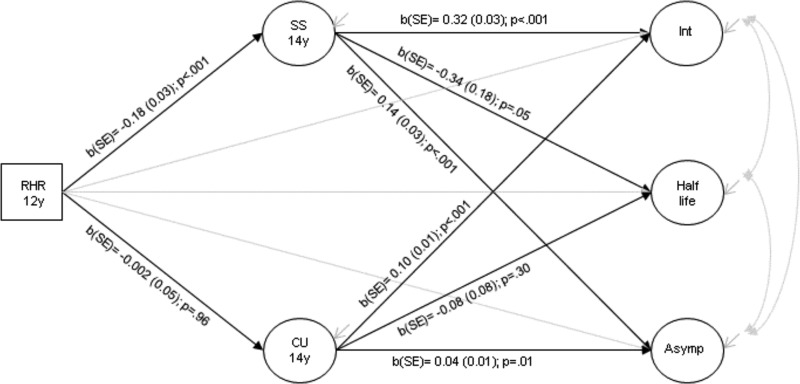
Structural model showing the direct effect of RHR on ASB growth factors and the indirect effects via sensation seeking and callous-unemotional traits: sociodemographic confounders adjusted for but not shown in the diagram: RHR, resting heart rate; SS, sensation seeking; CU, callous-unemotional traits; Int, intercept: Asymp, asymptote; circles represent latent variables and squares represent observed variables *N* = 4046.

Figure 3 shows results from the structural model examining the direct effect of RHR at age 12 years on the intercept, half-life and asymptote for ASB, and the indirect effects through sensation seeking and callous-unemotional traits at age 14 years. After adjusting for sociodemographic confounders, lower RHR was associated with higher levels of sensation seeking [*b*(s.e.) = −0.18 (0.03); *p* < 0.001] but not callous-unemotional traits [b(s.e.) = −0.002 (0.05); *p* = 0.96]. Additionally, higher sensation seeking was associated with both increased baseline levels [b(s.e.) = 0.32 (0.03); *p* < 0.001] and final levels of ASB [b(s.e.) = 0.14 (0.03); *p* < 0.001]. There was also weak evidence for an association between sensation seeking and the rate of decrease of ASB across adolescence [b(s.e.) = −0.34 (0.18); *p* = 0.05]. Higher callous-unemotional traits were associated with both increased baseline levels [*b*(s.e.) = 0.10 (0.01); *p* < 0.001] and final levels of ASB [*b*(s.e.) = 0.04 (0.01); *p* = 0.01], but not the rate of decrease.

Table 1 shows the total, direct and indirect effects of RHR on ASB growth factors. After adjusting for socio-demographic confounders, there was evidence for a total effect of RHR on baseline ASB levels in mid-adolescence [*b*(95% CI) = −0.08 (−0.14 to −0.02)], that was almost entirely explained through sensation seeking [indirect effect: *b*(95% CI) = −0.06 (−0.08 to −0.04)]. There was also evidence for an indirect effect of RHR on final levels of ASB via sensation seeking [*b*(95% CI) = −0.03 (−0.04 to −0.01)].

**Table 1.** Total, direct and indirect effects of RHR on ASB growth factors; showing unstandardised coefficient (95% confidence intervals); *N* = 4046

	Adjusting for sociodemographic confounders^a^			Adjusting for all confounders^b^		
	ASB intercept	ASB half-life	ASB asymptote	ASB intercept	ASB half-life	ASB asymptote
Total effect	−0.08 (−0.14 to −0.02)	−0.13 (−0.48 to 0.22)	−0.01 (−0.08 to 0.06)	−0.01 (−0.05 to 0.04)	−0.05 (−0.35 to 0.25)	0.00 (−0.05 to 0.05)
Indirect effect via SS	−0.06 (−0.08 to −0.04)	0.06 (−0.00 to 0.13)	−0.03, (−0.04 to −0.01)	−0.01 (−0.03 to −0.003)	0.02 (−0.01 to 0.05)	−0.01 (−0.01 to −0.00)
Indirect effect via CU	0.00 (−0.01 to 0.01)	0.00 (−0.01 to 0.01)	0.00 (−0.004 to 0.003)	−0.00 (−0.01 to 0.01)	0.00 (−0.01 to 0.01)	0.00 (−0.004 to 0.003)
Direct effect	−0.02 (−0.08 to 0.04)	−0.19 (−0.58 to 0.19)	0.02 (−0.06 to 0.09)	0.01 (−0.04 to 0.05)	−0.07 (−0.37 to 0.23)	0.01 (−0.05 to 0.06)

Note: ASB: antisocial behaviour; SS: sensation seeking; CU: callous-unemotional traits.

a socio-demographic confounders include household crowding index, housing tenure, maternal education, and ethnicity.

b additional confounders include child factors at approximately age 11 years (sex, age, BMI, diastolic blood pressure, medication use, the frequency of vigorous activity, alcohol and cigarette use) and parent factors (crime and alcohol problems).

After additionally adjusting for the child and parent-related confounders, all effects weakened. However, there was still evidence of a weak indirect effect of RHR, via sensation seeking, on both baseline ASB levels [*b*(95% CI) = −0.01 (−0.03 to −0.003)] and final ASB levels [*b*(95% CI) = −0.01 (−0.01 to −0.00)]. There were no direct effects of RHR on ASB growth factors before or after adjusting for confounders.

### Secondary analyses

**Fig. 4.**
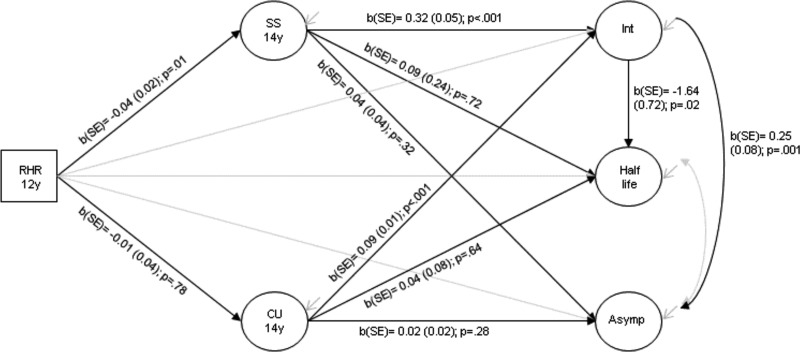
Structural model showing the direct and indirect effects of RHR on ASB growth factors after taking account of the effect of ASB intercept on ASB half-life and asymptote, sociodemographic, child and parent-related confounders adjusted for but not shown in the diagram: RHR, resting heart rate; SS, sensation seeking; CU, callous-unemotional traits; Int, intercept; Asymp, asymptote; circles represent latent variables and squares represent observed variables *N* = 4046.

Analyses were then performed to examine whether lower RHR was associated, via sensation seeking, with the slower decline and higher levels of ASB in adulthood, after accounting for ASB levels in mid-adolescence. Figure 4 shows the direct and indirect effects of RHR on ASB growth factors after taking account of the effect of the ASB intercept on the ASB half-life and asymptote. There was no evidence of an association between sensation seeking [*b*(s.e.) = 0.04 (0.04); *p* = 0.32] or callous-unemotional traits [*b*(s.e.) = 0.02 (0.02); *p* = 0.28] with final levels of ASB, after accounting for baseline ASB levels in mid-adolescence and all potential confounders. Additionally, there was no evidence of an association between sensation seeking or callous-unemotional traits with the rate of decrease of ASB across adolescence. Total, direct and indirect effects are shown in online Supplementary Table S4.

### Sensitivity analyses

Sensitivity analyses were then performed in order to assess the impact of missing data by re-running the fully-adjusted mediation model using IPW. Online Supplement 2 shows that conclusions from weighted analyses were the same.

## Discussion

### Summary of findings

In this UK population-based sample, lower RHR in childhood had an effect, via sensation seeking, on higher levels of ASB in mid-adolescence after adjusting for a range of psychiatric, socio-demographic and physical confounding factors. There was also initial evidence for an indirect effect of lower RHR on ASB levels in early adulthood, via sensation seeking, but this effect weakened substantially after accounting for potential confounders and ASB levels in mid-adolescence. Additionally, lower RHR had little impact on the rate of decrease of ASB across adolescence.

### Strengths and limitations

The main strengths of this study include the use of a large, representative, prospective sample with data available for both males and females across childhood, adolescence and early adulthood including information on a wide range of sociodemographic, parent- and child-related confounders. However, the findings need to be considered in the context of several limitations. First, as with most cohort studies, there was selective attrition over time. Proportionally few cohort members provided data on all measures across adolescence and early adulthood. However, all analyses were performed using FIML estimation which allowed over 4000 participants to be included; and the inclusion of auxiliary variables related to missing data (such as socio-demographic factors) or using IPW (inverse probability weighting) made little difference to the results. Second, there are other factors, not examined here, such as fearlessness or cognitive factors, which may be additional mechanisms for the association between low RHR and ASB; however, it is likely that these factors are not completely independent from sensation seeking. Additionally, given evidence for shared genetic risk for heart rate, sensation seeking and ASB, the role of genetic factors is important to consider (Ortiz & Raine, 2004; Baker et al. 2009; Portnoy & Farrington, 2015; Mann et al. 2017). Third, although a wide range of potential confounders were considered, we were not able to directly adjust for fitness level (only a proxy for fitness – frequency of vigorous physical activity). Finally, although there was evidence for an effect of RHR on ASB, via sensation seeking, the effect sizes were very small indicating that, in this sample, there may be other factors that are important to consider in explaining the course of ASB.

### Comparison with previous studies

In the current study, the association between lower RHR at age 11 years and baseline levels of ASB at age 15 years were almost entirely explained through sensation seeking at age 14 years. These findings support previous cross-sectional (Portnoy et al. 2014) and longitudinal (Sijtsema et al. 2010) studies that have identified sensation seeking in adolescence as a mechanism. However, both previous studies found a direct effect of heart rate on ASB not explained through sensation seeking, which was not the case here. This contradictory finding may be due, in part, to different aspects of sensation seeking, or ASB, being captured across studies, cross-cultural differences or the cross-sectional design and use of a male-only sample in previous research. Additionally, the total effect of RHR on ASB levels in mid-adolescence was substantially reduced after accounting for child-related confounders. This reduction was mainly due to adjusting for child sex, with males having both a lower heart rate and higher levels of ASB and sensation seeking.

There was also no evidence of an association between RHR in childhood and callous-unemotional traits in adolescence. This finding is in contrast to other studies that have found a cross-sectional association between RHR and child-report callous-unemotional traits in children (Gao et al. 2017) and male adolescents (Kavish et al. 2017), and a recent meta-analysis reporting evidence of an association between RHR and psychopathy (Portnoy & Farrington, 2015). The lack of association here could be due, in part, to using parent-report measures of callous-unemotional traits or examining effects over a longer time period. Additionally, variability in heart rate may be important to consider in relation to callous-unemotional traits.

### The course of ASB across adolescence and early adulthood

The present study expands on the extant literature by examining the role of sensation seeking and callous-unemotional traits in explaining the association between RHR and the persistence of ASB into adulthood. Although, there was evidence for an indirect effect of RHR on ASB levels in early adulthood, via sensation seeking, this effect was mainly explained through ASB levels in mid-adolescence. Additionally, RHR had little effect on the rate of decrease in ASB across adolescence. This finding supports previous results from a twin sample that showed no association between lower RHR at age 9 years and change in ASB between ages 9 and 14 years (Baker et al. 2009), and from a community sample of adolescent males that showed no association between RHR at age 16 years and desistance of ASB between age 17 and 20 years (Loeber et al. 2007). These findings highlight the difficulty in making long-term predictions regarding ASB desistance (Kazemian et al. 2009).

### Conclusions and implications

Lower heart rate in childhood was associated with ASB levels in mid-adolescence, indirectly via increased sensation seeking. Understanding the mechanism involved in the association between heart rate and ASB is important in order to best design interventions for ASB in those with a low heart rate (Portnoy & Farrington, 2015). These findings indicate that interventions for ASB in those with a low heart rate should target sensation seeking and teach young people prosocial ways to increase their autonomic activity (Ortiz & Raine, 2004; Wilson & Scarpa, 2011; Portnoy & Farrington, 2015).

## Supplementary material

For supplementary material accompanying this paper visit http://dx.doi.org/10.1017/S0033291717003683. click here to view supplementary material
