# Supplementary material for: Low resting heart rate, sensation seeking and the course of antisocial behaviour across adolescence and young adulthood
Source: Psychol Med. 2018 Jan 9;48(13):2194–201. doi: 10.1017/S0033291717003683 (PMC6533639; doi:10.1017/S0033291717003683)
Supplement: Supplementary file 1 [file S0033291717003683sup001.zip › S0033291717003683sup001/Hammerton_Supplement 1.docx]

**Supplement 1.** Detail on the derivation of the exponential decay model

For constructs where non-linear change over time is hypothesised (as for ASB across adolescence and early adulthood), the most commonly used strategy is to add a quadratic term to the model. A trajectory model with a quadratic term (or additional higher order polynomial functions) is non-linear with respect to time (change over time does not follow a linear pattern); however, the target function is linear with respect to the parameters and random coefficients meaning that the model is straightforward to specify in a SEM framework (Grimm *et al.*, 2013). Specifically, the predicted score for a given individual at a particular time is simply a weighted sum of their individual intercept, slope and quadratic scores (Grimm *et al,* 2011).

However, for behaviours such as ASB, a quadratic model is not consistent with underlying theory, given that, for example, ASB decreases between adolescence and adulthood, and this decrease slows with time until it begins to level off in early adulthood. Exponential models of change are being increasingly utilised within fields related to education and learning (Blozis *et al*, 2007; Grimm *et al*., 2013; Kelley & Maxwell, 2008; Preacher & Hancock, 2015; Zhang *et al,* 2012); however, exponential change is also relevant for certain mental health domains. Although quadratic and exponential models may show similar patterns of change over short periods of time, over longer time periods, the quadratic curve will change direction and head toward positive/ negative infinity whereas the exponential curve will begin to level off and reach a plateau, a pattern of change which is much more realistic for many constructs including ASB. This plateau (the asymptote) can be estimated as part of the model and so allows the final levels of the construct of interest to be examined, alongside the initial levels (the intercept). Therefore, the exponential decay model not only allows more complex and theoretically relevant longitudinal change to be examined, but also enables estimation of growth parameters that are of greater interest for research questions focused on ASB desistance.

There are many variations on the equation describing exponential growth or decay:

| $y=\theta_{a}-\left( \theta_{i}-\theta_{a} \right)exp(-\theta_{r}t)$ | (1) |
| --- | --- |

Here $\theta_{i}$ represents the starting value (intercept), $\theta_{a}$ represents the horizontal asymptote, $\theta_{r}$ governs the rate of change and $t$ represents time in years with baseline set to zero. Here we opt to substitute the rate of change ($\theta_{r}$) for the half-life ($\theta_{h})$as proposed by a number of authors including Preacher and Hancock (2015) and Rausch (2004), although unlike previous studies we combine this with the intercept and asymptote as both are of substantive interest in the current study.

| $y=\theta_{a}-\left( \theta_{i}-\theta_{a} \right)\left( \frac{1}{2} \right)^{\frac{t}{\theta_{h}}}$ | (2) |
| --- | --- |

The exponential model is nonlinear in the sense of representing change over time but additionally, the parameters in the model are related in a nonlinear way. When the target function is nonlinear with respect to the random coefficients, Taylor Series Expansion is needed (for further detail on Taylor Series Expansion see (Grimm *et al*., 2011; Grimm *et al*., 2013; Preacher & Hancock, 2015)). Briefly, the partial derivative of the target function with respect to each growth parameter was taken so that the model could be expressed as a linear combination of latent variables. The partial derivatives with respect to each parameter of interest were:

$$\frac{\partial y}{\partial\theta_{i}}=-\left( \frac{1}{2} \right)^{\frac{t}{\theta_{h}}}$$

$$\frac{\partial y}{\partial\theta_{a}}=1-\left( \frac{1}{2} \right)^{\frac{t}{\theta_{h}}}$$

$$\frac{\partial y}{\partial\theta_{h}}=\left( \left( \frac{t}{{\theta_{h}}^{2}} \right)\left( \ln\left( \frac{1}{2} \right) \right)\left( \theta_{i}-\theta_{a} \right)\left( \left( \frac{1}{2} \right)^{\frac{t}{\theta_{h}}} \right) \right)$$

The linearized target function, shown below, was then specifiable using SEM.

$\tilde{y}=\mu_{a}-\left( \mu_{i}-\mu_{a} \right)\left( \frac{1}{2} \right)^{\frac{t}{\mu_{h}}}$ + ($\theta_{i}- \mu_{i})\left( -\left( \frac{1}{2} \right)^{\frac{t}{\mu_{h}}} \right)$ + ($\theta_{a}- \mu_{a})\left( 1-\left( \frac{1}{2} \right)^{\frac{t}{\mu_{h}}} \right)$

+ ($\theta_{h}- \mu_{h}) \left( \left( \frac{t}{{\mu_{h}}^{2}} \right)\left( \ln\left( \frac{1}{2} \right) \right)\left( \mu_{i}-\mu_{a} \right)\left( \left( \frac{1}{2} \right)^{\frac{t}{\mu_{h}}} \right) \right)$

The exponential decay model was specified using a modified version of the SEM-based structured latent curve modelling (SLCM) approach for modelling non-linear trajectories (Preacher & Hancock, 2015). This method has been described in detail elsewhere (Preacher & Hancock, 2015); briefly, the means of the growth factors (intercept, half-life and asymptote) were set to zero and the intercepts of the repeated measures were constrained to equal the target function, in order to capture the mean trend. S1 Figure 1 shows example estimated exponential decay trajectories that differ on the intercept (Figure A), the half-life (Figure B) and the asymptote (Figure C).

**S1 Figure 1.** Example exponential decay trajectories showing impact of changes in growth factors; Figure A shows the impact of changing the intercept with the half-life and asymptote held constant; Figure B shows the impact of changing the half-life with the intercept and asymptote held constant; Figure C shows the impact of changing the asymptote with the intercept and half-life held constant

A B C


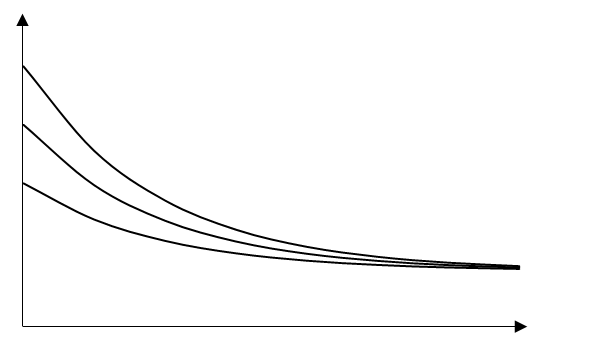

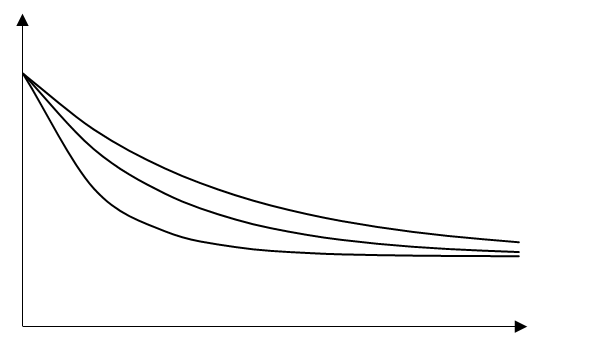

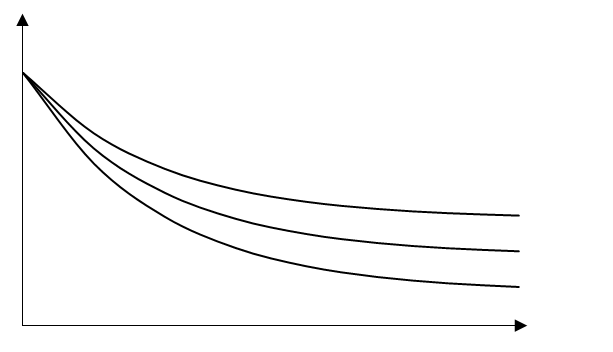


Increasing asymptote

Increasing half-life

Increasing intercept
