# Supplementary material for: Low resting heart rate, sensation seeking and the course of antisocial behaviour across adolescence and young adulthood
Source: Psychol Med. 2018 Jan 9;48(13):2194–201. doi: 10.1017/S0033291717003683 (PMC6533639; doi:10.1017/S0033291717003683)
Supplement: Supplementary file 1 [file S0033291717003683sup001.zip › S0033291717003683sup001/Hammerton_Supplement 2_revised.docx]

**Supplement 2.** Detail on the inverse probability weighting (IPW) used to address missing data

IPW has been recommended over alternative methods for dealing with missing data (such as multiple imputation) in situations where whole blocks of data are missing for a large proportion of individuals (Seaman *et al*., 2012). Weights were derived from a logistic regression analysis between a set of measures assessed in pregnancy that were independently predictive of missing data and/ or variables in the analysis (maternal smoking in pregnancy, marital status, parity, housing tenure and child gender) and inclusion in the final sample (*N* = 4,046/11,015). Minimal missing data on indicators used to derive weights were singly imputed as the modal or mean value (all indicators had < 6% of values missing). The Hosmer-Lemeshow test was used assess the fit of the missingness model, with results showing no indication of poor fit (Hosmer-Lemeshow χ^2^ (df) = 3.01 (8); *p* = 0.93). Weights ranged from 2.0 to 12.0.

The fully-adjusted mediation model was rerun using inverse probability weighting to address any potential bias caused by participant drop-out. Weighted estimates (shown in S2 Table 1) were similar to unweighted estimates.

**S2 Table 1.** Comparison of weighted (from IPW analyses) and unweighted estimates for total, direct and indirect effects of RHR on ASB growth factors; showing unstandardised coefficient (95% confidence intervals); *N* = 4,046

|  | Weighted estimates | | | Unweighted estimates | | |
| --- | --- | --- | --- | --- | --- | --- |
|  | ASB intercept | ASB half-life | ASB asymptote | ASB intercept | ASB half-life | ASB asymptote |
| Total effect | -0.01 (-0.07, 0.05) | -0.05 (-0.36, 0.26) | 0.003 (-0.06, 0.06) | -0.01 (-0.05, 0.04) | -0.05 (-0.35, 0.25) | 0.00 (-0.05, 0.05) |
| Indirect effect via SS | -0.01 (-0.02, 0.00) | 0.01 (-0.01, 0.03) | -0.003 (-0.01, 0.00) | -0.01 (-0.03, -0.003) | 0.02 (-0.01, 0.05) | -0.01 (-0.01, -0.00) |
| Indirect effect via CU | -0.00 (-0.01, 0.01) | 0.00 (-0.004, 0.01) | -0.00 (-0.004, 0.003) | -0.00 (-0.01, 0.01) | 0.00 (-0.01, 0.01) | 0.00 (-0.004, 0.003) |
| Direct effect | 0.00 (-0.06, 0.06) | -0.06 (-0.37, 0.26) | 0.01 (-0.05, 0.07) | 0.01 (-0.04, 0.05) | -0.07 (-0.37, 0.23) | 0.01 (-0.05, 0.06) |

Note: ASB: antisocial behaviour; SS: sensation seeking; CU: callous-unemotional traits; results adjusted for all potential confounders including sociodemographic factors assessed in pregnancy (child sex, household crowding index, housing tenure, maternal education, and ethnicity), child factors at approximately age 11 years (age, BMI, diastolic blood pressure, medication use, frequency of vigorous activity, alcohol and cigarette use) and parent factors (crime and alcohol problems
