# Supplementary material for: Low resting heart rate, sensation seeking and the course of antisocial behaviour across adolescence and young adulthood
Source: Psychol Med. 2018 Jan 9;48(13):2194–201. doi: 10.1017/S0033291717003683 (PMC6533639; doi:10.1017/S0033291717003683)
Supplement: Supplementary file 1 [file S0033291717003683sup001.zip › S0033291717003683sup001/Hammerton_Supplementary Figure 2_revised.docx]

**Supplementary Figure 2.** Flow chart of retention in the ‘Avon Longitudinal Study of Parents and Children’ (ALSPAC) sample

Initial sample after exclusions

*N* = 13,775

Data available for at least 1 of 4 repeated measures of ASB

*N* = 6,814

Pregnant women enrolled in ALSPAC Phase 1

*N* = 14,541

Complete data on confounders

*N* = 4,046

Exclusion criteria

Multiple births

Withdrawn from study

Sample used in main analyses

Invited to take part in at least 1 of 4 repeated assessments for ASB

*N* = 11,015

Sample used to derive weights

Offspring alive at one year

*N* = 13,988
