# Supplementary material for: Low resting heart rate, sensation seeking and the course of antisocial behaviour across adolescence and young adulthood
Source: Psychol Med. 2018 Jan 9;48(13):2194–201. doi: 10.1017/S0033291717003683 (PMC6533639; doi:10.1017/S0033291717003683)
Supplement: Supplementary file 1 [file S0033291717003683sup001.zip › S0033291717003683sup001/Hammerton_Supplementary references.docx]

# **Supplementary references**

**Blozis SA, Conger KJ, Harring JR** (2007). Nonlinear latent curve models for multivariate longitudinal data. *International Journal of Behavioral Development* **31**, 340–346.

**Grimm KJ, Ram N, Estabrook R** (2011). Nonlinear Structured Growth Mixture Models in Mplus and OpenMx. *Multivariate Behavioral Research* **45**, 887–909.

**Grimm K, Zhang Z, Hamagami F, Mazzocco M** (2013). Modeling nonlinear change via latent change and latent acceleration frameworks: Examining velocity and acceleration of growth trajectories. *Multivariate Behavioral Research* **48,** 117–143.

**Kelley K, Maxwell SE** (2008). Delineating the average rate of change in longitudinal models. *Journal of Educational and Behavioral Statistics* **33**, 307–332.

**Preacher KJ, Hancock GR** (2015). Meaningful aspects of change as novel random coefficients : A general method for reparameterizing longitudinal models. *Psychological Methods* **20**, 84–101.

**Rausch JR** (2004). *Designing longitudinal studies of negative exponential growth according to the reliabilities of growth parameter estimators*. Unpublished thesis, University of Notre Dame, Notre Dame, IN.

**Seaman SR, White IR, Copas AJ, Li L** (2012). Combining Multiple Imputation and Inverse-Probability Weighting. *Biometrics* **68**, 129–137.

**Zhang Z, McArdle JJ, Nesselroade JR** (2012). Growth rate models: emphasizing growth rate analysis through growth curve modeling. *Journal of Applied Statistics* **39**, 1241–1262.
