# Supplementary material for: Low resting heart rate, sensation seeking and the course of antisocial behaviour across adolescence and young adulthood
Source: Psychol Med. 2018 Jan 9;48(13):2194–201. doi: 10.1017/S0033291717003683 (PMC6533639; doi:10.1017/S0033291717003683)
Supplement: Supplementary file 1 [file S0033291717003683sup001.zip › S0033291717003683sup001/Hammerton_Supplementary Table 1_revised.docx]

**Supplementary Table 1a.** Confirmatory factor analysis (CFA) using ordinal sensation seeking items; *N* = 5,668

| Question | Standardised loading |
| --- | --- |
| 1. I like the feeling of standing next to the edge on a high place and looking down | 0.59 |
| 2. When I listen to music I like it to be loud | 0.24 |
| 3. I stay away from movies that are said to be frightening | 0.33 |
| 4. I like to ride on the roller coaster and other fast rides | 0.30 |
| 5. I would never gamble with money, even if I could afford it | 0.22 |
| 6. I like a movie where there are lots of explosions and car chases | 0.59 |
| 7. It would be interesting to see a car accident happen | 0.62 |
| 8. When the water is very cold, I prefer not to swim even if it is a hot day | 0.11 |
| 9. I think it would be exciting to be in a battle during a war | 0.74 |
| Variance | 1.71 (0.14) |

Note: items 3, 5 and 8 were reverse coded

**Supplementary Table 1b.** Confirmatory factor analysis (CFA) using ordinal callous-unemotional traits; *N* = 6,681

| Question | Standardised loading |
| --- | --- |
| 1. Child is genuine in their expression of emotions | 0.76 |
| 2. Child makes a good first impression but people change their minds after they get to know them | 0.61 |
| 3. Child has fast-changing emotions | 0.49 |
| 4. Child is genuinely sorry if they have hurt someone or acted badly | 0.74 |
| 5. Child can seem cold-blooded or callous | 0.74 |
| 6. Child keeps promises | 0.72 |
| Variance | 4.57 (0.29) |

Note: items 1, 4 and 6 were reverse coded
