# Supplementary material for: Low resting heart rate, sensation seeking and the course of antisocial behaviour across adolescence and young adulthood
Source: Psychol Med. 2018 Jan 9;48(13):2194–201. doi: 10.1017/S0033291717003683 (PMC6533639; doi:10.1017/S0033291717003683)
Supplement: Supplementary file 1 [file S0033291717003683sup001.zip › S0033291717003683sup001/Hammerton_Supplementary Table 2_revsied.docx]

**Supplementary Table 2a.** Descriptive statistics for RHR and ASB

|  | Males  (n = 1,837) | Females  (n = 2,209) | | Total  (N = 4,046) |
| --- | --- | --- | --- | --- |
|  | Mean (standard deviation) | | | |
| RHR (bpm; 12y) | 73.72 (10.68) | | 78.09 (10.73) | 76.11 (10.93) |
| ASB (number; 15.3y) | 0.97 (1.53) | | 0.57 (1.01) | 0.76 (1.30) |
| ASB (number; 15.7y) | 1.05 (1.50) | | 0.61 (1.07) | 0.81 (1.30) |
| ASB (number; 17.5y) | 0.41 (0.87) | | 0.21 (0.54) | 0.29 (0.71) |
| ASB (number; 18.1y) | 0.40 (0.84) | | 0.16 (0.50) | 0.27 (0.68) |
| ASB (number; 18.3y) | 0.43 (0.87) | | 0.25 (0.66) | 0.31 (0.75) |
| ASB (number; 19.1y) | 0.40 (0.79) | | 0.28 (0.74) | 0.33 (0.76) |
| ASB (number; 20.5y) | 0.44 (0.85) | | 0.19 (0.53) | 0.29 (0.68) |
| ASB (number; 21.3y) | 0.44 (0.80) | | 0.19 (0.54) | 0.29 (0.67) |

Note: RHR: resting heart rate; ASB: antisocial behaviour

**Supplementary Table 2b.** Descriptive statistics for potential confounders

|  | Males  (n = 1,837) | Females  (n = 2,209) | | Total  (N = 4,046) |
| --- | --- | --- | --- | --- |
| Sociodemographic (pregnancy) |  | |  |  |
| Housing tenure |  | |  |  |
| Privately rented; n (%) | 125 (6.8) | | 144 (6.5) | 269 (6.7) |
| Subsidised housing; n (%) | 103 (5.6) | | 158 (7.2) | 261 (6.5) |
| Maternal education |  | |  |  |
| High school only; n (%) | 631 (34.4) | | 772 (35.0) | 1403 (34.7) |
| No high school qualifications; n (%) | 296 (16.1) | | 386 (17.5) | 682 (16.9) |
| Crowding (>1 person per room); n (%) | 50 (2.7) | | 61 (2.8) | 111 (2.7) |
| Ethnicity (non-white); n (%) | 66 (3.6) | | 77 (3.5) | 143 (3.5) |
| Child-based (approximately age 12 years) |  | |  |  |
| Age (months); mean (SD) | 140.47 (2.41) | | 140.54 (2.48) | 140.51 (2.45) |
| Body mass index; mean (SD) | 18.79 (3.25) | | 19.19 (3.39) | 19.01 (3.33) |
| Diastolic blood pressure; mean (SD) | 58.18 (6.45) | | 59.04 (6.44) | 58.65 (6.46) |
| Any medication use; n (%) | 421 (22.9) | | 529 (24.0) | 950 (23.5) |
| Frequency of vigorous activity; mean (SD) | 2.70 (0.79) | | 2.44 (0.67) | 2.56 (0.74) |
| Drank alcohol before age 11; n (%) | 141 (7.7) | | 130 (5.9) | 271 (6.7) |
| Smoked a cigarette before age 11; n (%) | 37 (2.0) | | 41 (1.9) | 78 (1.9) |
| Parent-based (child’s birth to 11 years) |  | |  |  |
| Parental crime; n (%) | 236 (12.9) | | 285 (12.9) | 521 (12.9) |
| Problematic alcohol use; n (%) | 129 (7.0) | | 185 (8.4) | 314 (7.8) |

Note: SD: standard deviation
