# Supplementary material for: Low resting heart rate, sensation seeking and the course of antisocial behaviour across adolescence and young adulthood
Source: Psychol Med. 2018 Jan 9;48(13):2194–201. doi: 10.1017/S0033291717003683 (PMC6533639; doi:10.1017/S0033291717003683)
Supplement: Supplementary file 1 [file S0033291717003683sup001.zip › S0033291717003683sup001/Hammerton_Supplementary Table 3_revised.docx]

**Supplementary Table 3.** Means, variances and correlations for ASB growth factors; showing parameter estimate (standard error); *N* = 4,046

|  | 1. | 2. | 3. |
| --- | --- | --- | --- |
| 1. ASB intercept | 1 |  |  |
| 2. ASB half-life | -0.59 (0.15) | 1 |  |
| 3. ASB asymptote | 0.80 (0.29) | -0.24 (0.33) | 1 |
| Mean | 0.99 (0.03) | 1.46 (0.17) | 0.25 (0.03) |
| Variance | 1.90 (0.11) | 6.15 (3.59) | 0.16 (0.10) |

Note: ASB: antisocial behaviour
