# Supplementary material for: Low resting heart rate, sensation seeking and the course of antisocial behaviour across adolescence and young adulthood
Source: Psychol Med. 2018 Jan 9;48(13):2194–201. doi: 10.1017/S0033291717003683 (PMC6533639; doi:10.1017/S0033291717003683)
Supplement: Supplementary file 1 [file S0033291717003683sup001.zip › S0033291717003683sup001/Hammerton_Supplementary Table 4.docx]

**Supplementary Table 4.** Total, direct and indirect effects of RHR on ASB growth factors after taking account of the effect of ASB intercept on ASB half-life and asymptote; showing unstandardised coefficient (95% confidence intervals); *N* = 4,046

|  | Adjusting for sociodemographic confounders^1^ | | | Adjusting for all confounders^2^ | | |
| --- | --- | --- | --- | --- | --- | --- |
|  | ASB intercept | ASB half-life | ASB asymptote | ASB intercept | ASB half-life | ASB asymptote |
| Total effect | -0.08 (-0.14, -0.02) | -0.13 (-0.48, 0.22) | -0.01 (-0.08, 0.06) | -0.01 (-0.05, 0.04) | -0.05 (-0.35, 0.25) | 0.00 (-0.05, 0.05) |
| Indirect effect via SS | -0.06 (-0.08, -0.04) | -0.01 (-0.06, 0.04) | -0.01 (-0.02, -0.002) | -0.01 (-0.03, -0.003) | -0.00 (-0.03, 0.02) | -0.002 (-0.01, 0.002) |
| Indirect effect via CU | 0.00 (-0.01, 0.01) | 0.00 (-0.004,0.003) | 0.00 (-0.002, 0.001) | -0.00 (-0.01, 0.01) | 0.00 (-0.004, 0.003) | 0.00 (-0.002, 0.001) |
| Direct effect | -0.02 (-0.08, 0.04) | -0.22 (-0.59, 0.16) | 0.02 (-0.05, 0.09) | 0.01 (-0.04, 0.05) | -0.06 (-0.34, 0.22) | 0.004 (-0.05, 0.05) |

Note: ASB: antisocial behaviour; SS: sensation seeking; CU: callous-unemotional traits; ^1^socio-demographic confounders include household crowding index, housing tenure, maternal education, and ethnicity; ^2^additional confounders include child factors at approximately age 11 years (sex, age, BMI, diastolic blood pressure, medication use, frequency of vigorous activity, alcohol and cigarette use) and parent factors (crime and alcohol problems)
