# Supplementary figures and images for: Low resting heart rate, sensation seeking and the course of antisocial behaviour across adolescence and young adulthood
Source: Psychol Med. 2018 Jan 9;48(13):2194–201. doi: 10.1017/S0033291717003683 (PMC6533639; doi:10.1017/S0033291717003683)

**Supplementary Figure 1.** Timeline for data collection.


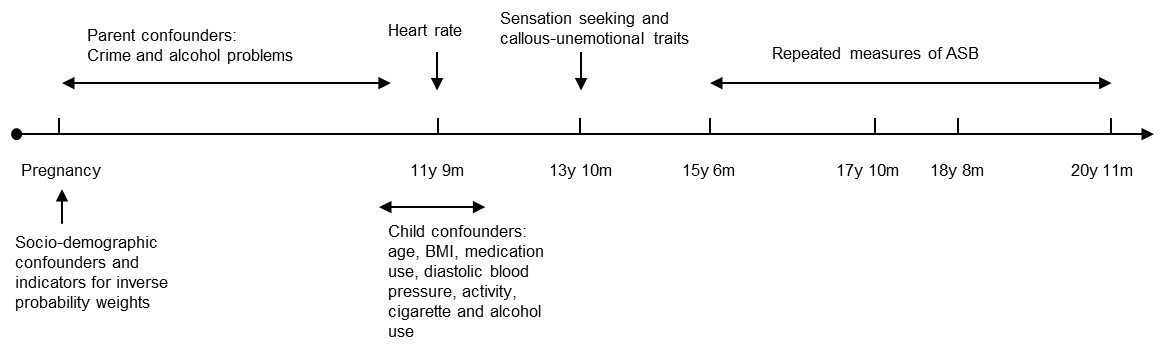

Supplement: Supplementary file 1 [file S0033291717003683sup001.zip › S0033291717003683sup001/Hammerton_Supplementary Figure 1_revised.docx]
